# Trends in Animal Rabies Surveillance in the Endemic State of Minas Gerais, Brazil

**DOI:** 10.1371/journal.pntd.0003591

**Published:** 2015-03-16

**Authors:** Misael E. Oviedo-Pastrana, Camila S. F. Oliveira, Renato O. Capanema, Rafael R. Nicolino, Teresa J. Oviedo-Socarras, João Paulo A. Haddad

**Affiliations:** 1 Federal University of Minas Gerais—UFMG, Veterinary School, Department of Preventive Veterinary Medicine, Belo Horizonte, Minas Gerais, Brazil; 2 Departamento de Sanidad Animal, Facultad de Medicina Veterinaria y Zootecnia, Universidad de Córdoba, Montería, Colombia; The Global Alliance for Rabies Control, UNITED STATES

## Abstract

Rabies is a viral zoonosis affecting mammal species and causes large economic losses. Included among the neglected diseases, it is still insufficiently addressed by governments and the international community, despite formal surveillance and control programs. This study used a dataset of 10,112 rabies diagnoses in animals provided by the Brazilian passive surveillance system from 2001 to 2012. The positivity rate of the tested samples was 26.4%, and a reduction in the total samples sent during the last six years was observed. The kernel density map indicated case concentration in the south region and a decrease in density of rabies cases in the second period studied (2007 to 2012). The directional trend of positive rabies diagnoses remained in the south region, as shown by the standard deviational ellipse. The spatial scan statistic identified three large clusters of positive diagnoses, one in the first period (2001-2006) and two in the second period (2007-2012), indicating an expansion of risk areas. The decrease in rabies cases from 2006 to 2012 does not necessarily reflect lower viral circulation or improvement in actions by epidemiological surveillance; this decrease could indicate a deficiency in epidemiological surveillance during the observation period due to the increase in the silent areas. Surveillance should maintain an increasing or constant number of tests during the years in addition to a reduction in the number of outbreaks of rabies, which would indicate a lower positivity rate. The findings in this study indicate deterioration in the effectiveness of the passive surveillance for rabies. The number of rabies cases, total number of tests performed and positivity rate are good indicators for evaluating passive surveillance. This paper can function as a guide for the assessment and improvement of the actions in passive surveillance of rabies.

## Author Summary

The approach proposed in this study can provide a valuable contribution to strengthen the efforts to reduce rabies incidence, a relevant public health problem worldwide. In Brazil during the period from 2001–2012, 129 human cases of rabies were notified, with 128 deaths. It is imperative to decrease rabies virus circulation both in domestic and wildlife species, which has been reinforced by many studies demonstrating that rabies incidence is often much higher than the official reports. This study used a dataset of 10,112 rabies diagnoses in animals provided by the Brazilian passive surveillance and applied spatial analysis in addition to many indicators to highlight high positivity rate areas and accurately identify silent areas and regions where underreporting occurs. This type of approach is vital to establish priorities for epidemiologic surveillance strategically and to precisely focus actions in the required regions. These spatial analysis techniques combined with other sources of information can also be used to evaluate the effectiveness of passive surveillance for rabies and improve the strategies already adopted by government programs to control and prevent the disease. We consider our results an important achievement in envisioning effectiveness in the process to eliminate rabies worldwide.

## Introduction

Rabies is a viral zoonosis due to a *Lyssavirus* infection associated with neurological expression due to encephalitis or meningoencephalitis. It is one of the oldest known infectious diseases in the world but remains a neglected zoonotic disease, insufficiently addressed by governments and the international community [1], [2].

Most countries in the Americas have been declared free of human cases of dog-transmitted rabies, there is now only notification of human rabies transmitted by dogs in Bolivia, Peru, Honduras, Haiti, Dominican Republic, Guatemala and some states in northern and northeastern Brazil [3].

The urban human rabies, transmitted by dogs and cats, has decreased from 73 cases in 1990 to 17 cases in 2003 in Brazil [4]. Currently, vampire bat-transmitted rabies is a major public health problem in the subtropical and tropical areas in the Americas, from Mexico to Argentina [5]. However, in terms of rabies cases transmitted by all species in the period from 2001–2012, 129 human rabies cases were notified [6]. Particularly, cases in which humans were bitten by bats have increased in Brazil [7].

The urban cycle, particularly including domestic dogs, has been controlled [2]. However, sylvatic cycles are expanding with an increasing number of diagnoses in species such as a fox *Cerdocyon thous* and a common marmoset *Callythrix jacchus* [8] in Brazil, but vampire bats also play a main role in rabies transmission [9]. Consequently, the occurrence of rabies virus in vampire bats is reflected by the cattle rabies incidence [10] [11].

There are three vampire bat species, *Desmodus rotundus*, *Diphylla ecaudata* and *Diaemus youngii*. Two species feed only on blood of wild birds, and one species, *D*. *rotundus*, feeds on livestock and could be a transmitter of *Lyssavirus* causing bovine paralytic rabies, a source of large economic losses [12], [13].

Outbreaks in livestock transmitted by vampire bats were first observed between 1906 and 1908 in the State of Santa Catarina in Brazil, when approximately 4000 cattle and 1000 horses and mules died due to paralytic rabies [12]. Since this episode, Brazil has applied measures to control the bat-transmitted rabies. A total of 9,277 rabies cases were reported in Brazil from 2002 to 2009 (88.0% in cattle, 10.0% in horses and 2.0% in other species) [14]. Currently, the use of warfarin to reduce the vampire bat population and the livestock vaccination against rabies are regularly conducted [12].

Rabies in Brazil cause large economic losses in the productive sector due to animal deaths and in the public sector through costs in surveillance and control programs [15], [16]. Brazil has 211.28 million bovines, the second largest herd in the world, and the country also contains other herbivore species. Minas Gerais State has the largest equine population, the second largest cattle population, and the highest milk production [17]; therefore, it can be used as a model to evaluate rabies surveillance in Brazil.

The Brazilian Program for Rabies Control in herbivores aims to prevent the disease in cattle by focusing on the control of vampire bats (*Desmodus rotundus*), strategic vaccination and active and passive epidemiological surveillance [18], [12]. However, cases due to bat-transmitted rabies are largely underreported. The aim of this paper was to understand the spatial and temporal distribution of the rabies cases and to analyze this information to confront the robustness of these results with the effectiveness of passive surveillance to identify trends in disease behavior and the dynamics of the surveillance.

## Materials and Methods

### Study area

**Fig 1:**
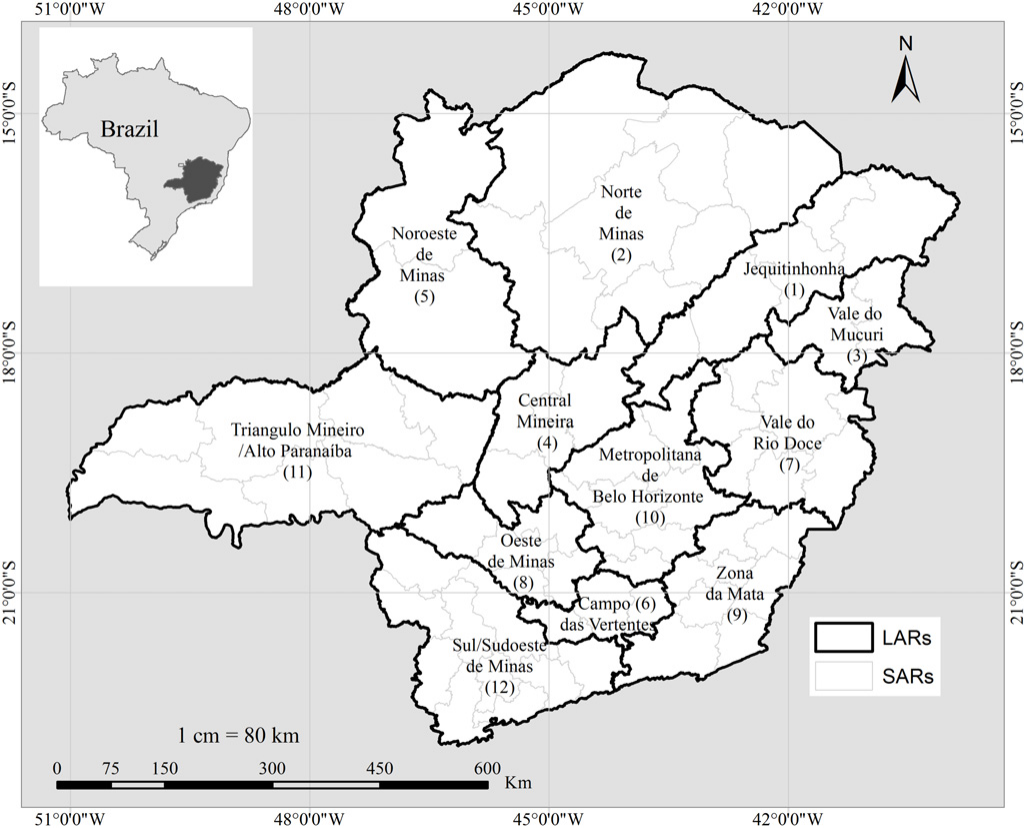
Minas Gerais State, regional geopolitical divisions and location in Brazil.

Minas Gerais is located in the southeastern region of Brazil, has an area of 586,528 Km^2^, and includes 853 municipalities, politically grouped in 66 small aggregate regions (SARs) and 12 large aggregate regions (LARs) [19]. SARs and LARs were adopted as basic areas for this study (Fig. 1) to facilitate the analysis interpretation.

### Data

This study was developed using data from the government agency responsible for animal health in the State (Instituto Mineiro de Agropecuária—IMA), which covers diagnoses from animals suspected of rabies between 2001 and 2012. The samples originated from all regions of the State and were sent voluntarily by farmers or by both private and public veterinarians. The variables in the database were animal species, year, month, location (municipality) and the rabies test results.

The results were geo-referenced using ArcGIS 9.3 [20], digital map files from the Brazilian Institute of Geography and Statistics (IBGE) with the political administrative borders of the large aggregate regions (LARs), small aggregate regions (SARs) and municipalities [19].

The rabies surveillance database in the GIS platform was used to map the distribution of the disease and also applied other spatial and temporal analysis. To identify trends in the disease behavior and the dynamics of the surveillance, tables and graphs were used to describe these patterns. The study was divided into two sub periods to highlight patterns; the first study period was from 2001 to 2006, and the second was from 2007 to 2012.

### Diagnoses

The samples sent to the government animal health laboratory were subjected to the direct immunofluorescence technique and to the biological proof (inoculation in mice or cells). Differential diagnoses were performed by histopathology and immunohistochemistry based on the guidelines of a specific manual with techniques for herbivore rabies control [18].

### Spatial and temporal analysis

A standard deviational ellipse, using 1 standard deviation, was calculated to find any directional trend among positive results in the municipalities considering all species. The Kernel density estimation (search radius = 100 Km) used to assess the intensity of positive results on the surface (km^2^), which allowed us to identify the areas of higher concentration of cases along the smoothed surface that was generated, using ArcGIS version 9.3.1 [20].

The space-time scan statistical analysis (Poisson model) to identify spatial clusters was applied only on herbivorous species in which it was possible to know its animal population (bovine, equine, ovine and caprine), the analysis was performed using SatScan version 9.2 [21], and the parameters were high rates, months as the time variable and the maximum spatial cluster size with upper limit 50% of the population at risk.

### Analysis of epidemiological surveillance

The studies on epidemiological surveillance of rabies involve three epidemiologic indicators: number of rabies outbreaks, the total number of tests performed and the relationship between positive tests and total tests (the positivity rate) using data from all species [22]. These indicators were selected due to the passive nature of the rabies data used to analyze surveillance in Minas Gerais State.

The relationship between positive results and the total tests performed was used to measure the reporting level of the rabies surveillance. Values equal or close to 1 express less surveillance actions, and values equal or close to 0 indicate an increase in surveillance activities [22]. This ratio was calculated for each municipality and for each month in the respective years, and the result was consolidated on a graph and represented on maps.

A projected epidemiological scenario was estimated based on the analysis of the best level of surveillance in the period studied; from 2001 to 2005, rabies surveillance achieved the recommended positivity rate without compromising the total number of tests performed. Therefore, the average positivity rate from 2001 to 2005 was used in the subsequent years in conjunction with the true total positive samples sent each year, resulting in the expected total of tests that should have been performed to ensure adequate surveillance in the projected scenario.

## Results

### Descriptive analysis

The passive surveillance system for rabies in Minas Gerais performed 10,112 rabies diagnoses between 2001 and 2012 (Table 1). Among the total results, 2,670 were positive, corresponding to 26.4% (25.5% to 27.2%, confidence interval of 95%), and 7,442 were negative. In addition to herbivore species, which are important in Brazilian livestock production, additional species such as swine, canine, feline, bats and other wild animals were also included in the analysis (excluding only the analysis using the Poisson Model). The percentages of positive animals for each species were the following: bats (2.42%), bovine (40.6%), equine (28.6%), caprine (23.8), ovine (12.0%), swine (7.3%), canine (3.4%), feline (0.8%), and others (7.9%).

**Table 1:** Diagnoses of rabies for each species affected, according to data provided by the animal rabies passive surveillance data in Minas Gerais, Brazil, from 2001 to 2012.

	2001 to 2006				2007 to 2012				
Species	Samples			Number of municipalities	Samples			Number of municipalities	Total samples
	+	-	Subtotal		+	-	Subtotal		
Bat	40	1,689	1,729	104	13	448	461	50	2,190
Bovine	1,531	2,284	3,815	523	799	1,131	1,930	456	5,745
Equine	145	321	466	184	87	259	346	164	812
Caprine	4	13	17	9	1	3	4	4	21
Ovine	3	24	27	19	3	20	23	17	50
Swine	2	20	22	16	1	18	19	16	41
Canine	31	965	996	137	6	94	100	46	1,096
Feline	0	104	104	30	1	14	15	12	119
Other	3	24	27	13	0	11	11	5	38
Total	1,759	5,444	7,203	556	911	1,998	2,909	494	10,112

### Spatial distribution

Among the 853 municipalities in Minas Gerais, 361 (42.3%) did not have any rabies diagnoses in the 12 years of study, 212 (24.8%) of which have not sent samples for diagnoses; these municipalities were classified as silent areas for rabies (Table 2). Between 2001 and 2006, 384 municipalities (45.0%) diagnosed positive cases; 297 (34.8%) did not send samples, and of the 7,203 samples sent in the period, 1,759 (24.4%) were diagnosed as positive. From 2007 to 2012, 317 municipalities (37.2%) were positive and 359 (42.0%) did not send samples. Of the 2,909 samples in the second period, 911 (31.3%) were diagnosed as positive. Among the municipalities diagnosed as positive in the first period, 80 (20.8%) did not send samples in the second period of the study and were classified as silent areas.

**Table 2:** Number of municipalities testing animal samples and total number of sent samples in Minas Gerais, Brazil, from 2001 to 2012.

Periods	Municipality with sample sent in the period?			Samples	
	Yes	No			% of positives
	Total (+)	Total	%	Total	
2001 to 2006	556 (384)	297	34.8	7,203	24.4
2007 to 2012	494 (317)	359	42.0	2,909	31.3
2001 to 2012	641 (492)	212	24.8	10,112	26.4

**Fig 2:**
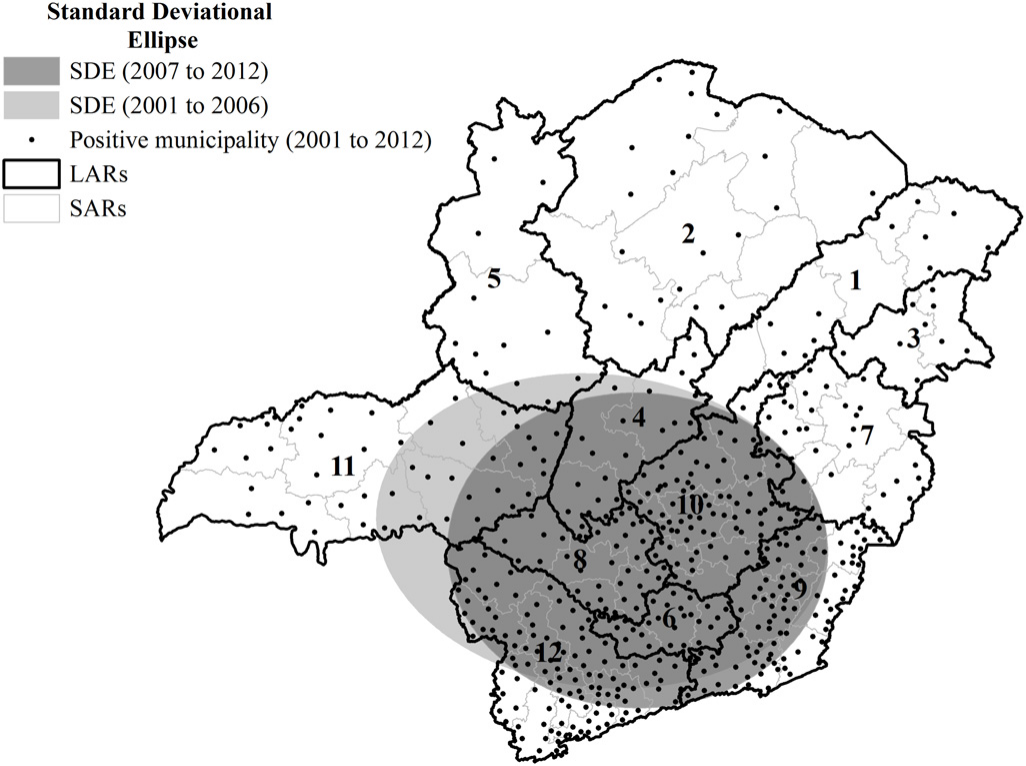
Standard deviational ellipse for positive diagnoses of animal rabies in all species in Minas Gerais, Brazil, from 2001 to 2012.

The standard deviational ellipse indicated a tendency in the south of the State, in the direction west-east (Fig. 2). As observed in the period from 2001 to 2006, the distribution was more concentrated in western Minas Gerais, mostly in the Triângulo Mineiro/Alto Paranaíba region (11). However, between 2007 and 2012, the distribution was more concentrated in the east.

**Fig 3:**
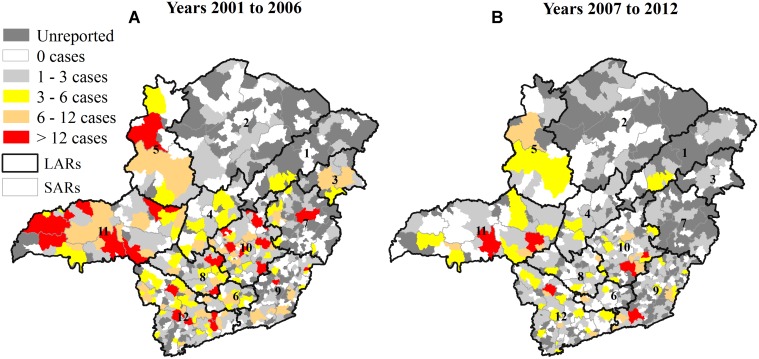
Spatial distribution of positive diagnoses of animal rabies in the first (A) and second (B) study periods by municipality in Minas Gerais, Brazil, from 2001 to 2012.

All regions reported cases of rabies, and fewer positive results were detected in northern and eastern Minas Gerais State (Fig. 3A and 3B). Four LARs contained 66% of the municipalities (140 of 212) that had not sent any samples in the studied period; 48 were located in LAR-2, 30 in LAR-1, 9 in LAR-3 and 53 inside the LAR-7. From 2001 to 2006, 41 SARs included 93 municipalities that showed at least six cases (Fig. 3A). From 2007 to 2012, only 22 SARs included 32 municipalities that reported at least six cases (Fig. 3B). The mean of cases reported in the first period was 1.24 cases/municipality/month, and 61 municipalities reported over 3 cases/month; in the second period, the mean was 1.13 cases/municipality/month, and 11 municipalities reported over 3 cases/month.

### Time tendency

**Fig 4:**
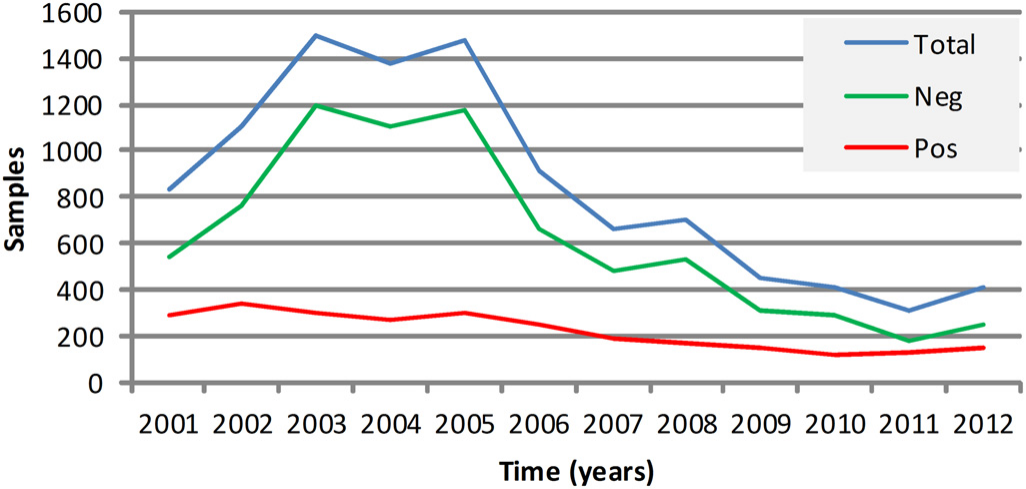
Overall trend of positive, negative and total animal rabies diagnoses performed in Minas Gerais, Brazil, from 2001 to 2012.

The trend of diagnoses over time is presented in Fig. 4. In the first period of the study, the positive results remained above 200 per year, with a mean of 293 cases per year. The negative results increased in the first three years and stabilized until 2005. In 2006 and afterwards, the negative results decreased considerably, and the mean number of negative results was 907 per year. In the second period of the study, a different pattern was found in the results; the positive results were below 200 per year, and the mean was 151 cases per year. The negative result presented an accentuated decreased tendency, and the mean number of negative diagnostics was 333 tests/year.

### Spatial and temporal analysis

**Fig 5:**
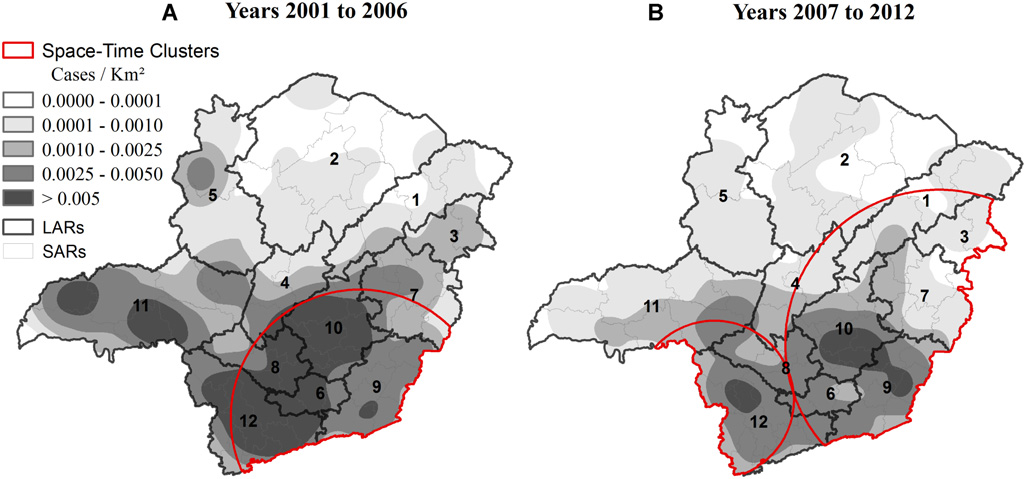
Kernel density map and spatial-temporal clusters of positive diagnoses for herbivore rabies in Minas Gerais, Brazil, from 2001 to 2012.

The kernel density map (Fig. 5) showed a high concentration of cases in the southern region of the State during both testing periods. However, the case concentration reduced in the second period. The spatial-temporal analysis with Poisson model (Fig. 5) identified a cluster in the southern region in the first period of the study. In the second period, two clusters were identified, including southern and eastern Minas Gerais. The kernel density map presented a reduction in the case concentration of rabies during the second period. However, the spatial scan statistic recognized an increase in the rabies risk area. The LARs with high concentration of cases and cluster areas in the second period (using the two techniques) were the following: Jequitinhonha (1), Vale do Mucuri (3), Vale do Rio Doce (7), Metropolitana de Belo Horizonte (10), Zona da Mata (9), Oeste de Minas (8), Campo das Vertentes (6) and Sul/Sudoeste de Minas (12).

### Epidemiologic surveillance analysis

**Fig 6:**
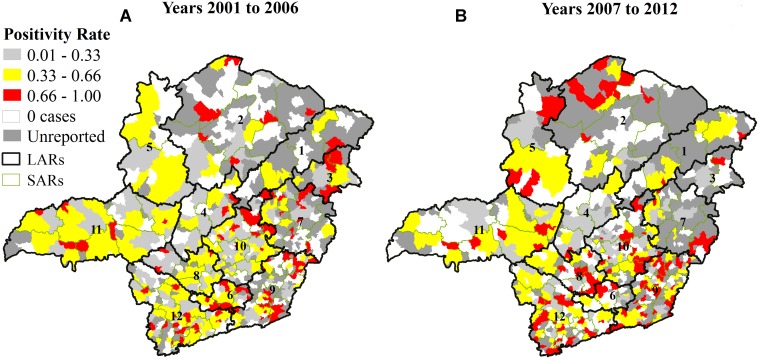
Spatial representation of the positivity rate of animal rabies in Minas Gerais, Brazil, from 2001 to 2012.

The positivity rate of rabies diagnoses is spatially represented for each municipality in Fig. 6. In the second period of the study (Fig. 6B), areas with high positivity rates (above 0.66) and areas with no reports of the disease increased compared to the first period (Fig. 6A).

**Fig 7:**
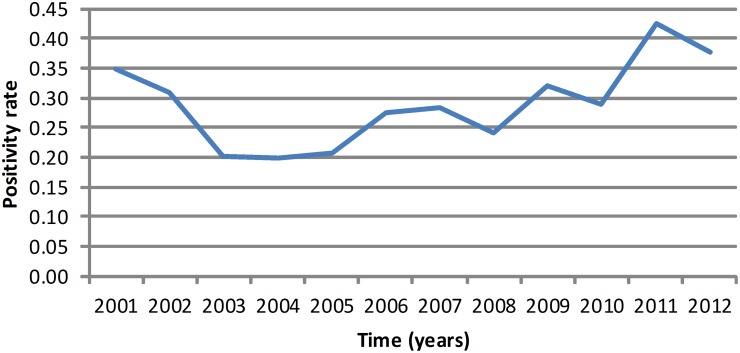
Overall trend of the positivity rates for animal rabies diagnoses in Minas Gerais State, Brazil, from 2001 to 2012.

**Fig 8:**
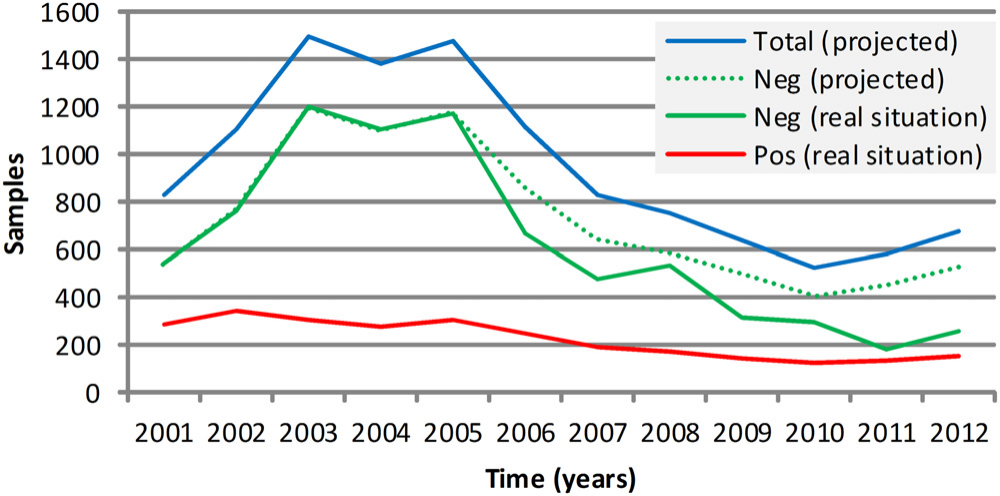
Projected epidemiological scenario of animal rabies in Minas Gerais, Brazil, from 2006 to 2012 estimated from the positivity rate of the first study period (2001 to 2005).

The positivity rate of rabies diagnoses from 2001 to 2012 is also represented in Fig. 7, synthesizing the behavior of the passive surveillance over time. Until 2005, the indicators were favorable for rabies surveillance. In contrast, from 2006 to the end of the study, there was an increase in the positivity rate and a reduction in total samples sent. In Fig. 8, the projected scenario was presented to ensure adequate rabies surveillance, considering the epidemiologic indicators from 2001 to 2005, number of rabies outbreaks, the total number of tests performed and the positivity rate.

## Discussion

Comparing both periods in this study (2001 to 2006 and 2007 to 2012), the positive diagnoses in all species and the total number of samples sent decreased in the second period; bovines, equines, bats and canines were the animals with the greatest decrease in positive diagnoses (Table 1). Barbosa *et al*. [8] indicated this trend regarding only canine and feline rabies surveillance in Minas Gerais from 2000 to 2006. In canines and felines, a reduction of approximately 90% in tested samples could be due to the success of the Urban Rabies Control Program. However, in this situation, it is important to guarantee that the actions of the passive surveillance system for urban rabies in the State are appropriate. Menezes et al. [23] reported a decrease in the total number of tests performed for bovine rabies from 1998 to 2006 in Minas Gerais; this reduction was considered to be due to improved surveillance, rabies mass vaccination and efficiency of programs to control vampire bat populations. However, the authors also found an increase in municipalities with underreporting problems.

In comparing canine and feline results in the Urban Rabies Control Program, reductions of 73% in bat tests, 50% in bovine tests, 26% in equine tests, 76% in caprine tests and 15% in ovine tests were not explained by the actions of the Herbivore Rabies Control Program. In the second period of the study, there was an increase in bovine population. Therefore, we expected a concomitant rise in rabies surveillance with an increase in number of tests [22], although the number of tests decreased.

Table 1 reveals the decrease in the number of positive and negative diagnoses comparing the two periods. The decrease in the number of positive results is observed in the map of spatial distribution of rabies (Fig. 3) as well; in the second period, many SARs reduced the number of positive diagnoses with large difference between the north/northeast and other regions of Minas Gerais, which does not represent absence of the disease. Instead, this difference indicates silent areas where underreporting probably occurs. However, Silva *et al*. [15] did not mention the occurrence of rabies in some regions of Minas Gerais in the time range considered (1976 to 1997).

Low socioeconomic development, low cattle density and the absence of bat shelters could be raised as potential explanations for regions with no reports (Fig. 6), but research involving socioeconomic analysis of Minas Gerais LARs shows municipalities with low socioeconomic development both in the north and south regions [24]. According to Silva *et al*. [15], Minas Gerais has climatic conditions and shelters to maintain bat populations. Regarding cattle density, the four LARs located in the north (1, 2, 3 and 5) cover 44.5% of the territory [19] and account for 33.7% of the cattle population [17], but the LARs accounted for only 7.5% of the total cases (2,670) identified in the 12 years under study considering all species (bovines accounted for 87.3% of rabies cases). The introduction of domestic animals, deforestation of the rain forest (“Mata Atlântica”) biome and urbanization contributed to the occurrence of rabies across the country [8], [23].

A reduction in the detected number of rabies cases is desirable if the total number of tests performed remains stable. Fig. 4 presents the decrease in the total tests performed from 2005 to the end of the study.

The kernel density map (Fig. 5) verified a decrease in density of rabies cases in the second period (2007 to 2012), with the directional trend of rabies positive diagnoses remaining in the south of the State, as shown in the standard deviational ellipse (Fig. 2). The decrease in rabies cases does not necessarily reflect lower viral circulation or improvement of actions by epidemiological surveillance. Instead, this finding could indicate a deficiency in epidemiological surveillance.

In Fig. 6B, an overall increase in areas with higher positivity rates is shown compared to Fig. 6A, suggesting a decrease in the passive surveillance in Minas Gerais. In Fig. 7, it is possible to see this problem evolving over time, presenting a favorable scenario until 2005 and a deterioration from 2006 to 2012 (Fig. 4). This new scenario is even more complex due to the increase in municipalities that do not report epidemiological information about rabies, although most municipalities have viral circulation. The identification of rabid bats or with *Lyssavirus* in their saliva and bat shelters depends essentially on active surveillance, although most actions of Brazilian animal rabies surveillance are passive. The lack of infrastructure and resources for collecting and submitting samples is often a greater barrier to rabies surveillance than lack of diagnosing facilities as considered by Halliday *et al*.[25].

The projected epidemiological scenario, from 2005 to 2012, was designed according to the suitable epidemiologic indicators from 2001 to 2005 (Fig. 8) and allowed us to determine the number of negative tests and the total number of tests that should be performed in accordance with the number of positives cases found. The expected number of tests was 3,883 negative diagnoses and 5,014 total diagnoses, an increase in 31.2% and 24.2%, respectively. Rabies incidence is often much higher than the official reports [26], [27].

The definition of a projected scenario in terms of surveillance should consider a comprehensive period of time, and the indicators should undergo comparison analysis due to their intrinsic relationships. Surveillance should maintain an increasing or constant total number of tests over the years in addition to the reduction in the number of rabies cases, which indicates a lower positivity rate [22]. An improvement in epidemiological surveillance can be presented with a lower positivity rate if it is mostly due to the reduction in the number of positive diagnoses.

### Conclusions

Rabies in Brazil is a complex situation and needs more research, and various socioeconomic and environmental factors need to be considered. Nevertheless, the method applied in this study allowed us to establish priorities for epidemiologic surveillance. The combined use of the total number of tests performed, number of rabies cases, and the positivity rate are good indicators to evaluate passive rabies surveillance. The approach in this paper using spatial analysis techniques combined with other sources of information serves as a guide for improving the actions in passive rabies surveillance. The method can also be used to evaluate the effectiveness of the actions by passive rabies surveillance and to improve the strategies already adopted by government programs to control and prevent the disease in Brazil and in other countries.
